# Effectiveness of aspirin in preventing deep vein thrombosis following proximal femoral fracture surgery in Japan

**DOI:** 10.1038/s41598-025-88493-4

**Published:** 2025-02-05

**Authors:** Takao Ohmori, Kazukiyo Toda, Takuya Taoka, Takeshi Ishihara, Yasuo Ito

**Affiliations:** Department of Orthopaedic Surgery, Japanese Red Cross Kobe Hospital, 1-3-1, Wakinohamakaigandori, Kobe, Chuo-Ku 651-0073 Japan

**Keywords:** Proximal femoral fractures, Deep vein thrombosis, Aspirin, Prophylaxis, Surgery, Outcomes research, Fracture repair

## Abstract

Previous studies have shown that aspirin is effective as a prophylactic agent against venous thromboembolism (VTE) following proximal femoral fractures (PFF). In Japan, there is a lack of evidence regarding its efficacy and safety in this context. Consequently, aspirin is not covered by insurance for the prevention of venous thrombosis. This study aimed to investigate whether continued aspirin use in patients with PFF, who were already taking aspirin for cerebrovascular disease prevention before injury is effective as a prophylaxis for deep vein thrombosis (DVT). We retrospectively analyzed PFF patients (≥ 65 years) who underwent postoperative duplex ultrasonography from January 2010 to December 2023.The study compared patients taking aspirin alone (aspirin group) and those not taking antiplatelet agents or anticoagulants (control group), matched by propensity scores. We enrolled 1064 patients while 161 (15%) were in the aspirin group. After matching, 128 patients were analyzed. DVT incidence was not statistically significant between the aspirin (54) and control groups (60) (OR: 0.81; 95%CI: 0.49- 1.36; p = 0.44). Proximal DVT incidence was also similar (OR: 2; 95%CI: 0.50–7.00; p = 0.33). Additionally, since use of other postoperative antithrombotic prophylaxis (78%) is thought to have a significant impact on the incidence of DVT, a subgroup analysis was conducted to evaluate the effect of aspirin in patients who did not receive postoperative antithrombotic prophylaxis. Similarly, there was no statistically significant difference in either DVT (OR: 1.38; 95% CI: 0.55–3.42; p = 0.49) or proximal DVT (OR: 2.00; 95% CI: 0.37–10.92; p = 0.42). This study demonstrates that aspirin is not effective for preventing VTE in patients with PFF in Japan.

## Introduction

Venous thromboembolism (VTE) remains a significant concern, with a reported incidence of 11- 40% of patients after proximal femoral fractures (PFF) surgery^1,2^. Therefore, numerous clinical guidelines recommend postoperative thromboprophylaxis to reduce the risk of VTE associated complications and mortality^3–5^^.^Previous studies have shown that aspirin is effective as a prophylactic agent against VTE following PFF^6,7^. The American College of Chest Physicians Evidence-Based Clinical Practice Guidelines for the Prevention of VTE in orthopedic surgery patients recommend aspirin as a prophylaxis for VTE in PFF^3^. However, there is no evidence supporting the preventive effect of aspirin on VTE in patients with PFF in Japan, and the Japanese Orthopaedic Association guidelines do not recommend its use^8^. Consequently, aspirin is not used as venous thromboembolism (VTE) prophylaxis in these patients in Japan.

On the other hand, many patients routinely use aspirin for secondary prevention of cardiovascular and cerebrovascular events, particularly those with cardiovascular comorbidities^9^. Therefore, a considerable number of patients with PFF have already been taking aspirin prior to their injury. In our institution, PFF surgery is performed promptly without discontinuation of aspirin, and aspirin is continued postoperatively. This study aimed to investigate the efficacy of continued aspirin use as a prophylaxis for deep vein thrombosis (DVT) in patients with PFF, who were already taking aspirin for cerebrovascular disease prevention before injury.

## Materials and methods

### Study design and patient selection

We retrospectively collected data from PFF patients (aged ≥ 65 years) who underwent postoperative lower extremity duplex ultrasonography at our institution from January 2010 to December 2023. The postoperative lower limb duplex ultrasound was routinely performed on all patients whenever possible and was performed approximately one week after surgery. The patient demographics and characteristics were retrieved from the electronic patient database. The study compared patients who had been taking aspirin alone (aspirin group) and those who had not been taking antiplatelet agents or anticoagulants (control group) prior to their injury. Patients who discontinued aspirin preoperatively were excluded from the analysis. The study design was approved by the appropriate ethics review board. Informed consent was obtained from all patients and relevant persons, and no identifiable information of the participants is included in this study. This study was conducted following the tenets outlined in the Declaration of Helsinki.

### Outcomes and variables

The primary outcome was the incidence of DVT. The secondary outcome was the incidence of proximal DVT, recognized for its clinical significance due to the increased risk of Pulmonary embolism^10^. Proximal DVT was defined as a thrombus involving one or more central veins, including the popliteal, femoral, common femoral, profunda femoris, external iliac, internal iliac, and common iliac veins, as well as the inferior vena cava. The duration for observing the clinical outcomes was from the surgery to the period when the postoperative lower limb duplex ultrasound was performed.

Variables associated with VTE incidence were selected according to previous reports. The following variables were included: age, sex, body mass index (BMI), co-morbidities, American Society of Anesthesiology (ASA) score, time to operation, fracture and operation type, operative duration, intraoperative bleeding, blood transfusion requirement, time from operation to lower extremity duplex ultrasonography, and use of other postoperative antithrombotic prophylaxis. PFF were categorized into three groups: femoral neck, intertrochanteric, and subtrochanteric. Based on Garden classification^11^, femoral neck fractures were further subclassified into stable (stages 1 and 2) and unstable (stages 3 and 4) patterns. Additionally, Jensen classification^12^ was used to subdivide intertrochanteric fractures into stable (types 1 and 2) and unstable (types 3, 4, and 5) patterns.

Additionally, since use of other postoperative antithrombotic prophylaxis is thought to have a significant impact on the incidence of DVT, a subgroup analysis was conducted to evaluate the effect of aspirin in patients who did not receive postoperative antithrombotic prophylaxis.

### Prevention of VTE in PFF

Patients underwent preoperative rehabilitation, including automatic lower extremity movement and wore elastic stockings. Intraoperative and postoperative intermittent pneumatic compression devices were utilized. Patients were mobilized early, and those without contraindications were administered postoperative anticoagulants (Edoxaban, Enoxaparin, Fondaparinux) after 24 h postoperatively. All patients received the anticoagulants until postoperative lower extremity duplex ultrasonography examinations were performed. Patients diagnosed without DVT were discontinued administration anticoagulants. Patients diagnosed with DVT underwent follow-up approximately 1–2 weeks later to monitor thrombus enlargement on ultrasonography. Based on clinical findings, decisions were made regarding the continuation or discontinuation of anticoagulant therapy.

### Statistical analysis

Propensity score matching was used to identify patients with similar baseline characteristics. Propensity scores were estimated using a logistic regression model adjusted for patient characteristics. Matching was performed using 1:1 matching with a caliper width of 0.01 of the standard deviation of the logit of the propensity score, and without replacement. The incidences of DVT and proximal DVT in the aspirin and control groups were compared in matched pairs^13^. Logistic regression analyses were used to calculate unadjusted and adjusted odds ratios (ORs) for the incidences of DVT and proximal DVT. Continuous values are presented as median (interquartile range). Continuous and categorical variables were compared between the groups using Mann–Whitney U and chi-square tests with Yates’ correction, respectively. A two-tailed p-value < 0.05 was considered statistically significant. Statistical analyses were performed using R version 3.1.3 (The R Foundation for Statistical Computing, Vienna, Austria; www. R-project.org).

## Result

### Baseline characteristics

**Fig. 1:**
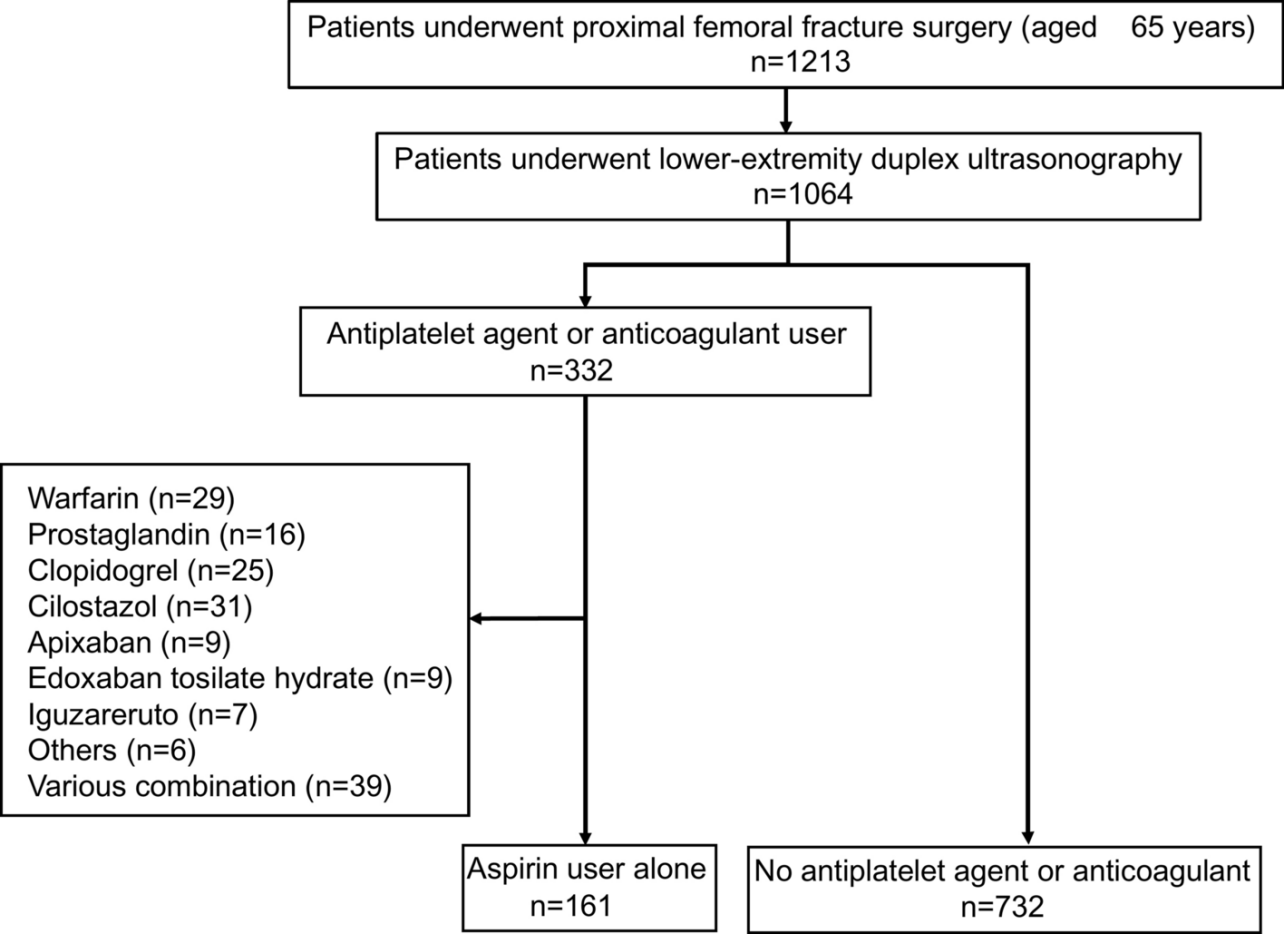
Patient disposition.

During the study period, a total of 1213 patients were enrolled. Of these, 1064 underwent lower-extremity duplex ultrasonography, and their data were available. A total of 332 patients (31%) were taking antiplatelet agents or anticoagulants, whereas 161 patients (15%) were administered aspirin only (aspirin group). All patients were administered 100 mg of aspirin daily. The duration of aspirin administration between surgery and the clinical outcomes was a median of 6 days (interquartile range, 4–7 days). The control group consisted of 732 patients not taking antiplatelet agents or anticoagulants (Fig. 1). Significant differences were observed in the age, co-morbidity (brain disease, cardiovascular disease, renal failure, diabetes mellitus, psychiatric disorder), ASA classification, blood transfusion requirements, and use of other postoperative antithrombotic prophylaxis between the two groups. Intraoperative blood loss was not significantly higher in the aspirin group. Postoperative antithrombotic prophylaxis was administered to a total of 697 patients (78%), with 102 patients (63%) in the aspirin group and 595 patients (82%) in the control group. and the duration of treatment was not significantly different between the two groups. (Table 1). A total of 347 patients (39%) were diagnosed with DVT, whereas 39 patients (4%) had proximal DVT. However, no significant differences were observed between the two groups in terms of the incidence of DVT (OR: 1.12; 95%CI: 0.79–1.58; p = 0.54) or proximal DVT (OR: 0.82; 95%CI: 0.34–2.00; p = 0.66) (Table 4).

**Table 1:** Baseline demographics and injury characteristics.

	All patients N = 893	Aspirin Group N = 161	Control Group N = 732	*p*-value
Age (years)	85 (79–90)	86 (81–90)	85 (79–89)	0.027
Male, n (%)	183 (20)	39 (24)	144 (20)	0.20
BMI* (kg/m^2^)	20 (18–23)	20 (18–22)	20 (18–23)	0.88
Co-morbidity, n (%)				
Brain disease	147 (16)	68 (42)	79 (11)	< 0.001
Cardiovascular disease	200 (22)	78 (48)	122 (17)	< 0.001
Pulmonary disease	81 (9)	14 (9)	67 (9)	1.00
Chronic liver disease	31 (3)	4 (3)	27 (4)	0.63
Renal failure	30 (3)	12 (7)	18 (3)	< 0.01
Diabetes mellitus	111 (12)	32 (20)	79 (11)	< 0.01
Personal history of cancer	102 (11)	17 (11)	85 (12)	0.79
Psychiatric disorder	182 (20)	18 (11)	164 (22)	< 0.01
Other disease	10 (1)	3 (2)	7 (1)	1.00
ASA**, n (%)				< 0.001
1	40 (4)	1 (1)	39 (5)	
2	551 (62)	69 (43)	482 (66)	
3	294 (33)	87 (54)	207 (28)	
4	8 (1)	4 (2)	4 (1)	
Fracture type, n (%)				0.91
Femoral neck				
Stable	161 (18)	26 (16)	135 (18)	
Unstable	284 (31)	51 (32)	233 (32)	
Intertrochanteric				
Stable	151 (17)	27 (17)	124 (17)	
Unstable	275 (31)	52 (32)	223 (30)	
Subtrochanteric	22 (3)	5 (3)	17 (3)	
Time to operation (day)	1 (0–3)	1 (0–2)	1 (0–3)	0.62
Operation, n (%)				0.89
Hip screw	142 (16)	22 (14)	120 (16)	
Side plate (Dynamic hip screw)	113 (13)	19 (12)	94 (13)	
Short intramedullary nail	278 (31)	54 (33)	224 (31)	
Long intramedullary nail	87 (10)	15 (9)	72 (10)	
Hemiarthroplasty	273 (30)	51 (32)	222 (30)	
Operation time (min)	67 (51–88)	68 (54–89)	67 (51–88)	0.71
Intra-operative bleeding (ml)	50 (10–100)	50 (10–110)	50 (10–100)	0.28
Blood transfusion requirement, n (%)	317 (35)	73 (45)	244 (33)	< 0.01
Time to postoperative ultrasonography (day)	6 (4–7)	6 (4–7)	6 (4–7)	0.60
Postoperative antithrombotic prophylaxis, n (%)				< 0.001
Enoxaparin	140 (16)	32 (20)	108 (15)	
Fondaparinux	18 (2)	4 (2)	14 (2)	
Edoxaban	539 (60)	66 (41)	473 (65)	
Duration of postoperative antithrombotic prophylaxis use (day)	5 (3–6)	5 (3–6)	5 (3–6)	0.99

*BMI; body mass index, **ASA; American Society of Anesthesiology ***DVT; deep vein thrombosis.

Continuous values are given as median (interquartile range).

### Propensity score matching analysis

A total of 128 patients were included in the analysis utilizing propensity score matching. The matching procedure considerably enhanced the balance of covariates in the matched cohort, resulting in similar characteristics for all elements of the propensity score between the groups (Table 2). The incidence of DVT (aspirin group, 54 patients vs, control group, 60 patients) are numerically reduced in patients taking aspirin, but these was not statistically significant (OR: 0.82; 95%CI: 0.49 -1.36; p = 0.44). Similarly, the incidence of proximal DVT (aspirin group, six patients vs. control group, three patients) was not statistically significant (OR: 2; 95%CI: 0.50 -7.00; p = 0.33) (Table 4). Aspirin use was not associated with a significantly higher risk of postoperative DVT or proximal DVT. Similarly, in the subgroup analysis of patients who did not receive postoperative antithrombotic prophylaxis, there was no statistically significant difference in either DVT (OR: 1.38; 95% CI: 0.55–3.42; p = 0.49) or proximal DVT (OR: 2.00; 95% CI: 0.37–10.92; p = 0.42) (Table 3, Table 4).

**Table 2:** Baseline characteristics after propensity score matching.

	All patients N = 256	Aspirin Group N = 128	Control Group N = 128	*p*-value
Age (years)	87 (81–90)	86 (81–90)	87 (83–91)	0.11
Male, n (%)	58 (22)	30 (23)	28 (22)	0.88
BMI* (kg/m^2^)	20 (18–22)	20 (18–22)	20 (18–22)	0.19
Co-morbidity, n (%)				
Brain disease	94 (37)	45 (35)	39 (30)	0.51
Cardiovascular disease	105 (41)	54 (42)	51 (40)	0.80
Pulmonary disease	28 (11)	12 (9)	16 (13)	0.55
Chronic liver disease	5 (2)	3 (2)	2 (2)	1.00
Renal failure	18 (7)	8 (6)	10 (8)	0.81
Diabetes mellitus	47 (18)	24 (19)	23 (18)	1.00
Personal history of cancer	28 (11)	15 (12)	13 (10)	0.84
Psychiatric disorder	32 (13)	15 (12)	17 (13)	0.85
Other disease	9 (4)	3 (2)	6 (5)	0.50
ASA**, n (%)				0.92
1	1 (0)	1 (1)	0 (0)	
2	116 (45)	59 (46)	57 (45)	
3	132 (52)	65 (51)	67 (52)	
4	7 (3)	3 (2)	4 (3)	
Fracture type, n (%)				0.49
Femoral neck				
Stable	41 (16)	22 (17)	19 (15)	
Unstable	73 (29)	39 (30)	34 (26)	
Intertrochanteric				
Stable	49 (19)	21 (16)	28 (22)	
Unstable	88 (34)	42 (33)	46 (36)	
Subtrochanteric	5 (2)	4 (4)	1 (1)	
Time to operation (day)	1 (0–2)	1 (0–2)	1 (0–2)	0.93
Operation, n (%)				0.89
Hip screw	36 (14)	19 (15)	17 (13)	
Side plate (Dynamic hip screw)	31 (12)	14 (11)	17 (13)	
Short intramedullary nail	91 (36)	44 (34)	47 (37)	
Long intramedullary nail	26(10)	12 (9)	14 (11)	
Hemiarthroplasty	72 (28)	39 (31)	33 (26)	
Operation time (min)	63 (52–87)	68 (53–89)	60 (51–83)	0.25
Intra-operative bleeding (ml)	50 (10–100)	50 (10–120)	50 (10–100)	0.60
Blood transfusion requirement, n (%)	121 (47)	57 (45)	64 (50)	0.45
Time to postoperative ultrasonography (day)	6 (4–7)	6 (4–7)	6 (4–7)	0.84
Postoperative antithrombotic prophylaxis, n (%)				0.13
Enoxaparin	51 (20)	27 (21)	24 (19)	
Fondaparinux	5 (2)	4 (3)	1 (1)	
Edoxaban	116 (45)	63 (49)	53 (41)	

*BMI; body mass index, **ASA; American Society of Anesthesiology.

Continuous values are given as median (interquartile range).

**Table 3:** Baseline characteristics excluding cases that received postoperative antithrombotic prophylaxis.

	Before propensity score matching			After propensity score matching		
	Aspirin Group N = 59	Control Group N = 137	p-value	Aspirin Group N = 37	Control Group N = 37	p-value
Age (years)	87 (84–91)	89 (83–92)	0.51	87 (84–90)	89 (84–93)	0.29
Male, n (%)	15 (25)	19 (14)	0.06	9 (24)	7 (20)	0.78
BMI* (kg/m^2^)	20 (18–22)	20 (18–23)	0.55	20 (19–22)	20 (18–23)	0.72
Co-morbidity, n (%)						
Brain disease	22 (37)	15 (11)	< 0.001	9 (24)	9 (24)	1.00
Cardiovascular disease	35 (59)	34 (25)	< 0.001	18 (49)	18 (49)	1.00
Pulmonary disease	8 (14)	13 (9)	0.45	4 (11)	4 (11)	1.00
Chronic liver disease	2 (3)	8 (6)	0.73	1 (3)	2 (6)	1.00
Renal failure	9 (15)	9 (7)	0.06	4 (11)	4 (11)	1.00
Diabetes mellitus	10 (17)	16 (12)	0.36	6 (16)	8 (22)	0.77
Personal history of cancer	4 (7)	17 (12)	0.32	3 (8)	4 (11)	1.00
Psychiatric disorder	5 (8)	27 (20)	0.06	4 (11)	3 (8)	1.00
Other disease	0 (0)	6 (4)	0.18	0 (0)	0 (0)	-
ASA**, n (%)			0.06			1.00
1	1 (2)	2 (2)		1 (3)	1 (3)	
2	24 (40)	81 (59)		15 (41)	15 (41)	
3	33 (56)	50 (36)		20 (53)	20 (56)	
4	1 (2)	4 (3)		1 (3)	0 (0)	
Fracture type, n (%)			0.61			0.44
Femoral neck						
Stable	10 (17)	17 (12)		6 (16)	4 (11)	
Unstable	16 (27)	42 (31)		10 (27)	10 (27)	
Intertrochanteric						
Stable	11 (19)	26 (19)		9 (24)	8 (22)	
Unstable	18 (30)	48 (35)		8 (22)	14 (37)	
Subtrochanteric	4 (7)	4 (3)		4 (11)	1 (3)	
Time to operation (day)	1 (1–2.5)	1 (0–3)	0.82	1 (1–2)	1 (0–2)	0.92
Operation, n (%)			0.93			0.85
Hip screw	9 (15)	19 (14)		6 (16)	4 (11)	
Side plate (Dynamic hip screw)	6 (10)	13 (9)		4 (11)	4 (11)	
Short intramedullary nail	18 (31)	48 (35)		11 (30)	15 (40)	
Long intramedullary nail	10 (17)	18 (13)		6 (16)	4 (11)	
Hemiarthroplasty	16 (27)	39 (28)		10 (27)	10 (27)	
Operation time (min)	71 (57–92)	67 (54–90)	0.66	70 (55–94)	63 (53–83)	0.65
Intra-operative bleeding (ml)	40 (10–100)	50 (10–100)	0.99	50 (10–100)	50 (10–100)	0.76
Blood transfusion requirement, n (%)	35 (59)	63 (46)	0.12	19 (51)	20 (54)	1.00
Time to postoperative ultrasonography (day)	6 (3–7)	6 (4–7)	0.39	6 (3–8)	6 (3–7)	0.94

*BMI; body mass index, **ASA; American Society of Anesthesiology, ***DVT; deep vein thrombosis.

Continuous values are given as median (interquartile range).

**Table 4:** Incidence of DVT before and after propensity score matching.

	**Aspirin group** **n (%)**	**Control group** **n (%)**	**Odds ratio** **(95% confidence)**	***p*-value**
**Outcome**				
Before propensity score matching				
DVT*	66 (41)	281 (38)	1.12 (0.79–1.58)	0.54
Proximal DVT	6 (4)	33 (5)	0.82 (0.34–2.00)	0.66
After propensity score matching				
DVT	54 (42)	60 (47)	0.82 (0.49–1.36)	0.44
Proximal DVT	6 (5)	3 (2)	2.00 (0.50–7.00)	0.33
Subgroup analysis**				
Before propensity score matching				
DVT	24 (41)	63 (46)	0.81 (0.41–1.56)	0.53
Proximal DVT,	4 (7)	5 (4)	1.91 (0.37–9.26)	0.46
After propensity score matching				
DVT	17 (46)	14 (38)	1.38 (0.55–3.42)	0.49
Proximal DVT	4 (11)	2 (5)	2.00 (0.37–10.92)	0.42

*DVT; deep vein thrombosis.

**Subgroup analysis was conducted in patients who did not receive postoperative antithrombotic prophylaxis.

## Discussion

We investigated the efficacy of continued aspirin use as a prophylaxis for DVT in patients with PFF, who were already taking aspirin for cerebrovascular disease prevention before injury. The results showed that the continuation of aspirin did not reduce the incidence of DVT or proximal DVT, and no effect on DVT prophylaxis was observed.

Aspirin inhibits platelet aggregation^14^; arterial thrombosis is a platelet-predominant phenomenon, often associated with atherosclerotic damage. In contrast, venous thrombosis is generally considered a disorder of plasma coagulation. Venous thrombi are fibrin-rich and originate from areas with slower blood flow, such as the deep veins of the legs. Platelets are less abundant than arterial thrombi. However, elevated levels of platelet and endothelial activation markers have been reported in patients with VTE, and the efficacy of aspirin in the primary or secondary prevention of VTE is biologically justified^15–18^.

Many studies have investigated the prophylactic effect of aspirin on VTE in orthopedic surgery. While some studies highlighted aspirin’s efficacy, others found it to be inferior to other agents^19,20^, emphasizing the necessity for further exploration, particularly for hip or knee arthroplasty. On the other hand, studies have reported that aspirin is as effective as other drugs, and there are also reports of significantly reduced mortality in orthopaedic trauma patients^21,22^. Few studies have examined the prophylactic effects of aspirin on PFF. One such report is the Pulmonary Embolism Prevention Trial^6^, which showed that aspirin predominantly prevented VTE. However, a VTE prophylaxis other than aspirin has not yet been described, and the effects of aspirin alone are unclear. Recent studies have examined the effectiveness of VTE prophylaxis in patients with femoral neck fractures undergoing hip arthroplasty and reported superior reductions compared to other agents^7^. On the other hand, the review analysis of VTE risk in patients with hip and lower limb injuries, including eight studies and 17,698 patients, showed that aspirin significantly did not reduced the risk of DVT compared to placebo (relative risk (RR): 0.47; 95%CI: 0.05–2.65)^23^. While several studies have reported that aspirin is an effective prophylactic agent for VTE, there have been skeptical evaluations and an identification of a lack of high-quality randomized controlled trials supporting its use in this context, necessitating further research^24,25^.

Furthermore, most studies on the effects of aspirin have examined its efficacy in treating symptomatic DVT prophylaxis. Therefore, the number of cases was limited (0.7 ~ 4.7%)^6,7,19–21^. However, asymptomatic DVT is present in 11–40%^1,2^. This study examined asymptomatic DVT by performing postoperative lower limb duplex ultrasound, which we believe is a more accurate examination of DVT incidence.

Regarding the bleeding risk of aspirin, this study found no significant difference in intraoperative blood loss between the groups, although the aspirin group showed a higher tendency for postoperative transfusion requirement. However, this finding needs to be adjusted for patient backgrounds. Previous reports^6,26^ on aspirin use in PFF suggest that the bleeding risk is generally low, indicating that aspirin can be used relatively safely in this context.

These results provide evidence that supports determining whether aspirin is effective as VTE prophylaxis in PFF surgeries in Japan. Although the present study did not demonstrate the efficacy of aspirin for DVT prevention following surgery for PFF, aspirin is widely recommended in many countries due to its lower risk of bleeding and cost-effectiveness. If future studies confirm its efficacy, the use of aspirin alone for thromboprophylaxis may offer significant clinical and economic benefits. Further research specifically investigating the use of aspirin for VTE prevention in Japan is warranted.

The main limitation of our study is that aspirin was used for the secondary prevention of cardiovascular and cerebrovascular events in patients with cardiovascular comorbidities, but not for the prevention of VTE. Additionally, this was a retrospective single-center survey. Further multicenter randomized controlled trials in which aspirin is administered to prevent VTE in Japan are needed. The aspirin dose used in this study was 100 mg daily. Aspirin as a prophylaxis for VTE has been reported in doses of 81–650 mg daily^6,7,19–22^, and the dose may affect the results. However, there are reports of no difference in the incidence of symptomatic VTE after THA with low-dose (81 mg) compared with standard-dose aspirin (325 mg)^27^.

## Conclusion

In patients with PFF who were taking aspirin prior to injury, the continuation of aspirin did not reduce the incidence of DVT or proximal DVT. This study demonstrates that aspirin is not effective for preventing VTE in patients with PFF in Japan.

## Supplementary Information

Supplementary Information.

## Data Availability

Data is provided within the manuscript or supplementary information files.
